# Supplementary figures and images for: Neuroprotection in early stages of Alzheimer’s disease is promoted by transthyretin angiogenic properties
Source: Alzheimers Res Ther. 2021 Aug 24;13:143. doi: 10.1186/s13195-021-00883-8 (PMC8385857; doi:10.1186/s13195-021-00883-8)

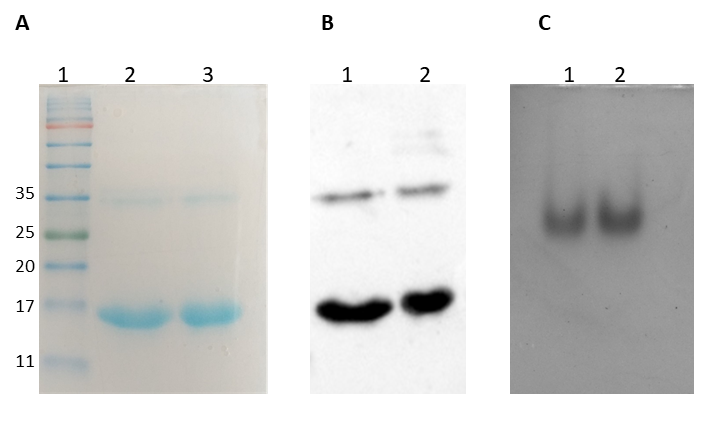

Supplement: Supplementary file 1 — Additional file 1: Figure S1. Analyses of the human recombinant TTR produced in the bacterial expression system. A- SDS-PAGE analysis of two different batches (lanes 2 and 3) showing the monomer as the main form. The dimer is also detected at a much lower extent. Lane 1 refers to the protein standards used and the values refer to the respective molecular weight. B- Western blot analysis of the two different batches (lanes 1 and 2) confirms the identity of TTR. C- Native-PAGE analysis of one of the TTR batches, before (lane 1) or after detoxification (lane 2), shows that a single protein form is detected, corresponding to the tetramer [file 13195_2021_883_MOESM1_ESM.png]

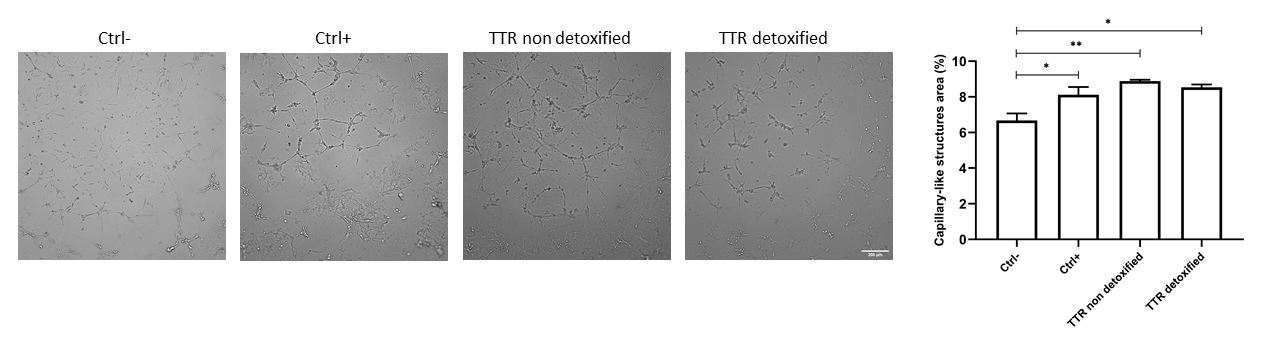

Supplement: Supplementary file 2 — Additional file 2: Figure S2. Tube formation by hCMEC/D3 cells. Representative images of tube formation by hCMEC/D3 cells. Cells were plated on Matrigel in the absence (negative control, Ctrl-) or presence (positive control, Ctrl+) of bFGF (35 ng/mL) or with 500 nM recombinant TTR, before (TTR non detoxified) or after detoxification (TTR detoxified). Scale bar = 200 μm. The quantification plot shows that treatment with TTR (either before or after detoxification) results in a significantly higher area covered by the capillary-like structures, than in the negative control. Data are expressed as mean ± SEM. * p < 0.05; ** p < 0.01 [file 13195_2021_883_MOESM2_ESM.png]
